# Genome sequence of *Bacillus pumilus* RI06-95 isolated during a *Microcystis* bloom in lake Champlain, USA

**DOI:** 10.1128/mra.00713-24

**Published:** 2024-08-30

**Authors:** Ashley Barkley, Kermisha Felix, Luke Myers, Nana Y. D. Ankrah

**Affiliations:** 1 Biological Sciences Department, State University of New York at Plattsburgh, Plattsburgh, New York, USA; 2 Lake Champlain Research Institute, State University of New York at Plattsburgh, Plattsburgh, New York, USA; Portland State University, Portland, Oregon, USA

**Keywords:** *Bacillus*, *Microcystis*, algal bloom, blue green algae

## Abstract

Here, we present the complete genome sequence of *Bacillus pumilus* RI06-95 isolated during a *Microcystis* bloom in Lake Champlain. The assembled genome comprises a 3.8 Mbp chromosome with a GC content of 42%, and two plasmids, pPZZ84 6.4 Kbp, GC content 37% and pSHB9 97Kbp, GC content 36%.

## ANNOUNCEMENT

The genus *Bacillus* represents a taxonomically diverse and metabolically versatile group of bacteria that inhabit a wide range of environments and perform important ecosystem functions (1). For example, in terrestrial systems, *Bacillus pumilus* is an important plant growth-promoting bacterium (2, 3), and in aquatic environments, *B. pumilus* has been demonstrated to have both growth-promoting (4) and inhibitory effects (5) on algae.

In this study, *B. pumilus* RI06-95 was isolated during a *Microcystis* bloom at Shelburne Bay, Lake Champlain (44.425833 N 73.232000 W), USA, in August 2021. Lake water was collected at 3 m depth using a 2L Van Dorn water sampler and inoculated on tryptic soy agar plates (Hardy Diagnostics). After incubating for 7 days at 25°C in the dark isolated colonies were inoculated onto fresh plates to generate pure cultures. Two additional rounds of re-streaking for isolation were performed prior to genomic DNA (gDNA)extraction.

A single bacterial colony was inoculated into tryptic soy broth (Hardy Diagnostics) and incubated at 25°C overnight to generate cultures for gDNA extraction. gDNA extraction from pure cultures, library preparation, sequencing, and genome assembly were performed by Plasmidsaurus (Eugene, OR, USA). gDNA was extracted using the Quick-DNA Fungal Bacterial Isolation Kit (Zymo Research). Sequencing libraries were prepared using Rapid Barcoding Sequencing Kit version SQK-RBK114.96 (Oxford Nanopore Technologies) with no fragmentation or size selection of DNA. Sequencing was conducted on the PromethION P24 platform (Oxford Nanopore Technologies), equipped with R.10.4.1 flow cells. Base calling was performed using Dorado v7.1.4 on super-accurate mode (Oxford Nanopore Technologies), yielding 43,920 reads; N50 read length 5,874 bp and a total sequence length of 164 Mbp.

Default parameters were used for all software unless otherwise specified. Reads were filtered with Filtlong v0.2.1 (https://github.com/rrwick/Filtlong) using a minimum Phred score of 10 and a minimum length of 1,000  bp. The genome was assembled using Flye v2.9.1 (6) and polished using Medaka v1.8.0 (https://github.com/nanoporetech/medaka). Genome overlap was identified and trimmed using Flye v2.9.1 (6), and the genome was not rotated. Assembly completeness was assessed using CheckM v1.2.2 (7). Genome annotation was performed using the NCBI Prokaryotic Genome Annotation Pipeline (8). A summary of assembly statistics and genome overview is provided in Table 1.

**TABLE 1:** Summary of assembly statistics and genome overview

Final genome coverage	39×
Plasmid pPZZ84 coverage	314×
Plasmid pSHB9 coverage	20×
CheckM completeness	99.59%
CheckM contamination	0.21%
Number of contigs	3
Total bp sequenced	163,581,533 bp
Total number of reads	43,920 reads
Longest read	48,807 bp
N50	5,874 bp
Genome size (bp)	3.8 Mbp
GC content (%) of genome	42%
Genes (total)	4,042
CDSs (total)	3,931
Genes (coding)	3,901
rRNAs	8, 8, 8 (5S, 16S, 23S)
tRNAs	82
ncRNAs	5

The genome sequence of *B. pumilus* RI06-95 is 3800112 bp (3.8 Mb) with a GC content of 42%. The genome contains 3,901 protein-coding genes and 82 tRNAs (Table 1). The genome of strain RI06-95 encodes many protein-coding genes predicted to be involved in the synthesis of various small bioactive compounds with antimicrobial and antifungal properties. antiSMASH v7.1.0 (9) analysis predicted 10 putative secondary metabolite biosynthetic gene clusters including regions with genes coding for non-ribosomal peptide synthetases and quorum sensing metabolites such as ranthipeptide. RI06-95 contains two plasmids pPZZ84 6,407 bp, GC content 37% and pSHB9 96,546 bp, GC content 36%. Plasmids were identified using Mash v2.3 (10) by screening against all plasmids available in the NCBI RefSeq database.

## Data Availability

The complete genome sequence of *Bacillus pumilus* RI06-95 was deposited in GenBank (accession: CP158181). The version described here is CP158181.1. The accession numbers for strain RI06-95 plasmids are CP158179 (pPZZ84) and CP158180 (pSHB9). The associated BioProject and BioSample accession numbers are PRJNA1119991 and SAMN41672263, respectively. Raw sequence reads have been submitted to the Sequence Read Archive (accession: SRR29542456).
